# Treating hypertrophic scar, post‐inflammatory hyperpigmentation, and post‐inflammatory hypopigmentation with intense pulsed light

**DOI:** 10.1111/srt.13823

**Published:** 2024-06-19

**Authors:** Lisa Kwin Wah Chan, Kar Wai Alvin Lee, Lee Cheuk Hung, Phoebe Kar Wai Lam, Jovian Wan, Massimo Vitale, Patrick Po‐Han Huang, Kyu‐Ho Yi

**Affiliations:** ^1^ EverKeen Medical Centre Hong Kong Hong Kong; ^2^ Perfect Skin Solution Hong Kong Hong Kong; ^3^ Asia‐Pacific Aesthetic Academy Hong Kong Hong Kong; ^4^ Private Practice Bologna Italy; ^5^ Huang PH Dermatology and Aesthetics Kaohsiung Taiwan; ^6^ Division in Anatomy and Developmental Biology Department of Oral Biology Human Identification Research Institute Yonsei University College of Dentistry Seodaemun‐gu Seoul South Korea; ^7^ Maylin Clinic (Apgujeong) Apgujeong Seoul South Korea

Dear Editor,

Hypertrophic scarring is a common complication following surgical procedures, trauma, or burns. These scars are often thick, and raised, and can be cosmetically and functionally problematic to the patient. Intense pulsed light (IPL) therapy has been known to effectively treat various skin conditions including vascular and pigmented lesions, but its use for hypertrophic scarring has been limited. This case report aims to present a case of successful IPL therapy in managing hypertrophic scars, demonstrating its potential as a safe and non‐invasive treatment option for this challenging condition.

A hypertrophic scar can sometimes spontaneously regress and does not spread to the tissues surrounding, it is elevated and visible. With continuous fibrosis and inflammation over a relatively long period, the scar can progress with extra deposition of collagen and fibroblast‐derived extracellular matrix proteins, together with dermal tissue proliferation. Different researchers proposed different treatments for the hypertrophic scar. Onion extract gel, silicone gel sheeting, laser, intralesional bleomycin, intralesional corticosteroid, intralesional interferon, pressure therapy, and surgical excision without or with grafting have been described in detail to treat the hypertrophic scar.^1^

Histologically, the hypertrophic scar is characterized by prominent blood vessels that are vertically oriented, with reticular and papillary dermis replacement in scar tissue and epidermal flattening.^2^ Sometimes a hypertrophic scar can be misdiagnosed as keloid, malignant dermatofibrosarcoma protuberans (DFSP) tumors, dermatofibroma, or lichen sclerosus.^3^

A 30‐year‐old Chinese female housewife presented with a right forearm scar for 3 years. She visited multiple general practitioner clinics and dermatology clinics and was told she could just leave it alone and the scar would improve. One general practitioner prescribed her topical steroid cream and she did not experience obvious improvement. She reported the scar was due to boiled water splashed on her forearm accidentally during her cooking at home around 3 years ago. It was a small wound at first, but it grew with discoloration of the surrounding skin in the 6 months after the accident. She was a non‐smoker non‐drinker, good past health.

Physical examination showed the patient had a 0.5 cm x 0.5 cm T‐shaped hypertrophic scar over the distal dorsal aspect of the right forearm, together with post‐inflammatory hyperpigmentation and post‐inflammatory hypopigmentation in the surrounding skin tissue. (Figure 1) The patient did not have other hypertrophic scars in other parts of her body. The patient only complained of pain just after the burn incident, he did not have any more discomfort once the scar appeared, he just felt unhappy with the look of the scar and wanted to have an improvement of the outlook. In this case, first, we choose to use IPL, as the IPL head is rectangular in shape and a lot bigger than the laser beam treatment area, this can facilitate our treatment area to be consistently treated with the same energy throughout the whole treatment area. Second, if we use lasers to treat this single lesion, we need three different laser machines to target hypertrophic scar, hyperpigmentation, and hypopigmentation respectively, in this case, we just need one IPL machine to treat three different modalities.

**FIGURE 1:**
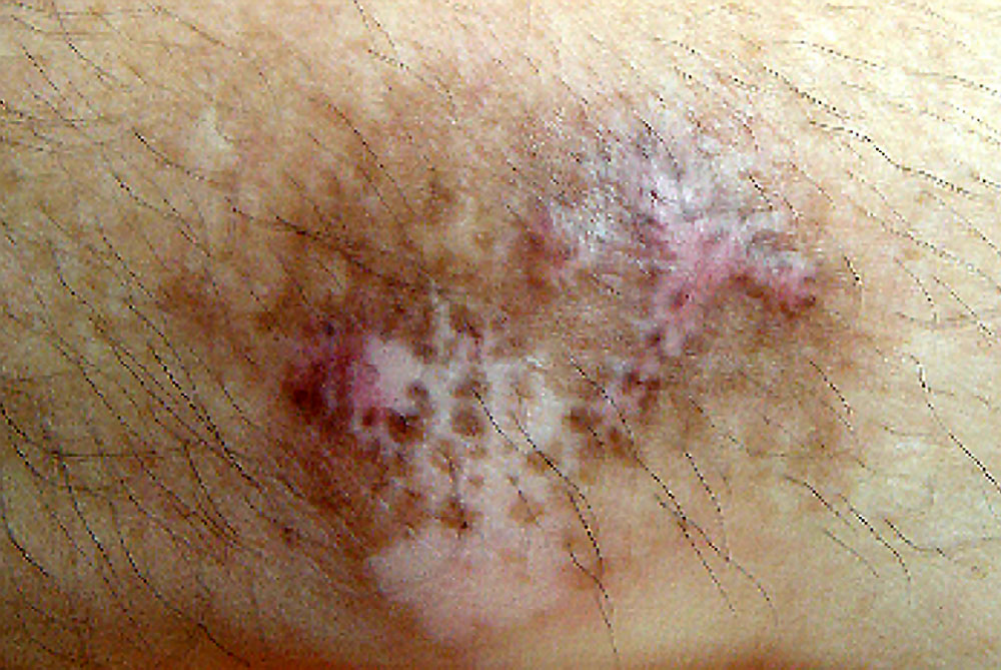
Hypertrophic scar before 1st IPL treatment. IPL, intense pulsed light.

The patient had 12 IPL treatments (we used the Lumenis M22 OPT machine in this case). Treatment parameters are summarized in Table 1.

**TABLE 1:** Intense pulsed light treatment parameters used are summarized.

Parameter used for the treatment	
Cutoff filters	560 nm, 590 nm, 640 nm, 695 nm
Energy output	10–40 J/cm^2^
Pulse width	3–100 ms
Pulse delay (thermal relaxation time)	1–120 ms
Triple pulses	590 nm, 640 nm, and 695 nm filters
Double pulses	560 nm and 640 nm filters
Duration	20–25 min

Before IPL treatment, patients are requested to wash their face with a neutral solution 10 min before photos are taken in the same treatment room under consistent lighting conditions, ensuring no make‐up or skin care products are present. Photographs are taken before each session from the front, both sides laterally, and obliquely, aiding in the evaluation of treatment results. During IPL treatment, patients are provided with shower caps to protect their hair and wear eye shields (corneal shields are not required), keeping their eyes shut throughout the procedure. The treatment area is evenly covered with a 5‐mm thick layer of chilled (4°C) colorless coupling gel. The immediate skin response is checked after three adjacent pulses on the test area, with fluency and/or relaxation time adjusted accordingly. Initial treatment settings are low and safe, with gradual power increases in small steps with each successive treatment while observing for any severe post‐treatment erythema. IPL treatment is recommended to be performed 7–15 times at intervals of 3–4 weeks without the need for topical anesthetic cream or general anesthesia, relying solely on cold gel applied to the treatment area. Post‐procedure care includes moisturizer, sunblock application, and advice on sun avoidance. The interval between treatments is 3 to 4 weeks, aligning with the usual skin turnover timeline of 21 to 28 days, as well as the standard time interval for IPL treatments.

The treatment was conducted every 3 to 4 weeks, with moisturizers given after each procedure. No intralesional steroid was injected. We also asked the patient to have strict sun avoidance to avoid post‐inflammatory hyperpigmentation. No intralesional steroid injection was used.

The clinical photos have been taken each time before the treatment. After the twelfth IPL Treatment, the patient was satisfied with the clinical result (Figure 2). We followed up with the patient and took other clinical photos 38 weeks after the twelfth treatment (Figure 3), there is no recurrence of the hypertrophic scar, and the patches of post‐inflammatory hyperpigmentation and post‐inflammatory hypopigmentation disappeared, which is the bonus of this treatment.

**FIGURE 2:**
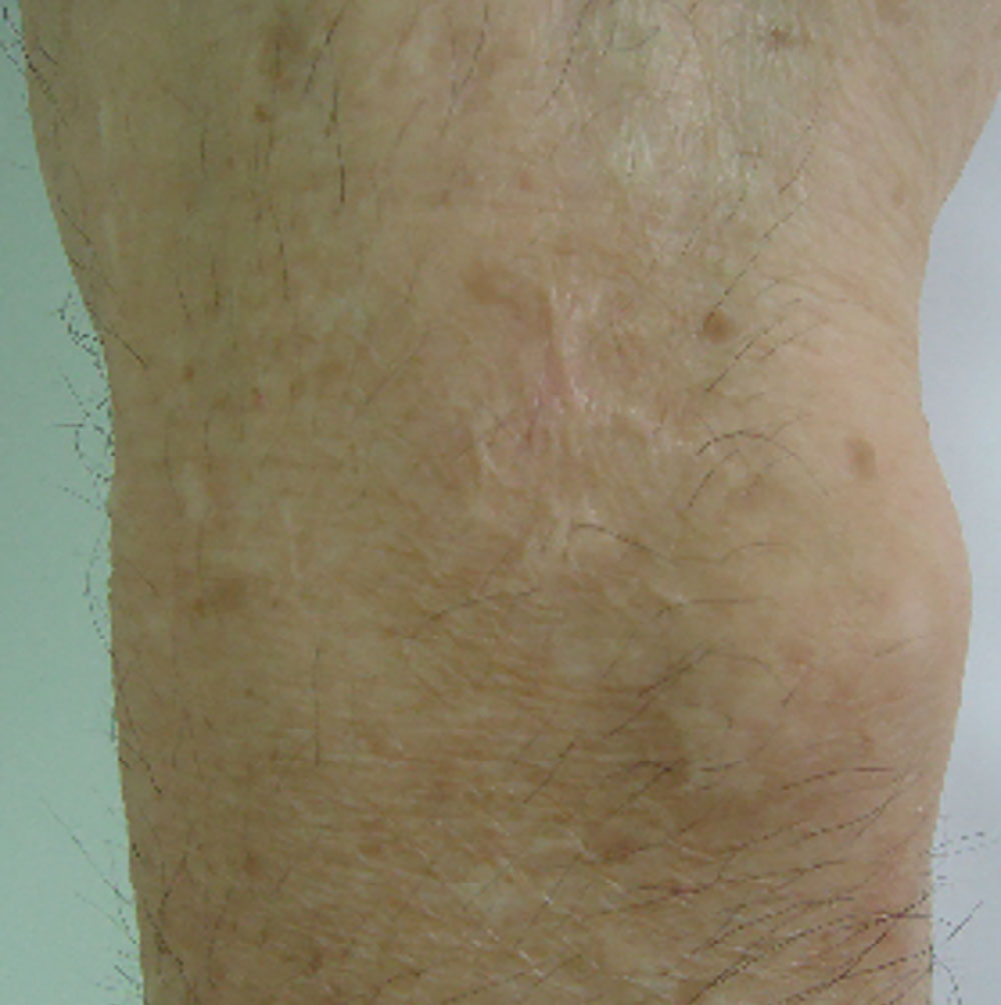
Hypertrophic scar after 12th IPL treatment (38 weeks later). IPL, intense pulsed light.

**FIGURE 3:**
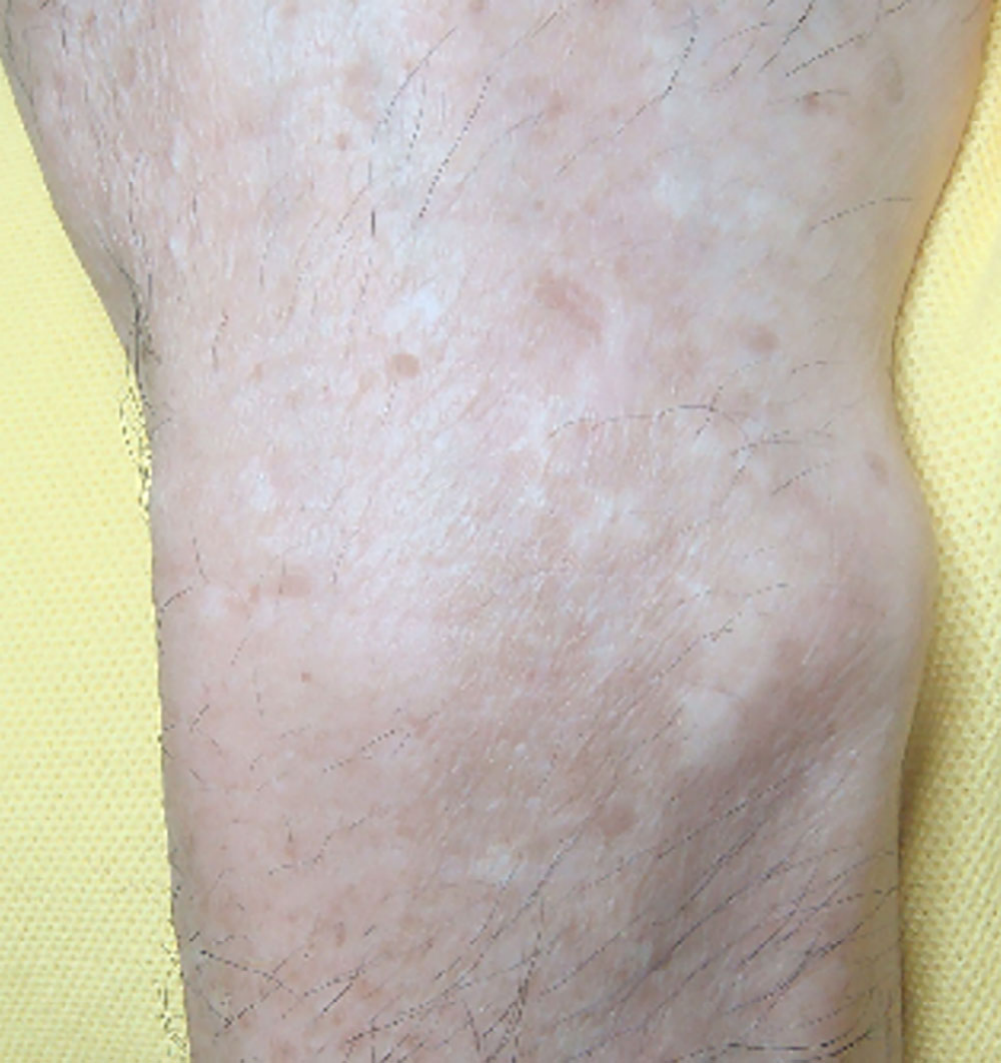
Hypertrophic scar after 12th IPL treatment (76 weeks later). IPL, intense pulsed light.

IPL involves the application of a non‐coherent, non‐laser broadband, filtered flash lamp source directed to the skin. Modification of various parameters allows flexibility in treatment. IPL was demonstrated to be effective in hair removal, inflammatory skin diseases like acne vulgaris and rosacea, pigmentation disorders like melasma, and skin photo rejuvenation. Erol et al.^4^ enrolled 109 hypertrophic scar patients in their study to test the efficacy of IPL on the hypertrophic scar. They received 8 treatments on average at 2 to 4 weeks intervals. The team showed good clinical improvement in hardness, erythema, height reduction, and scar appearance in most of the patients (92.5%).

Our case report has long follow‐up and high‐resolution pre‐treatment, on‐treatment, and post‐treatment clinical photos to demonstrate the progress of hypertrophic scar improvement. Furthermore, the post‐inflammatory hyperpigmentation and post‐inflammatory hypopigmentation surrounding the area were also improved with recovery of normal skin color after the whole treatment course. We did not use intralesional steroids to improve the scar, which provided an alternative treatment option for patients who have steroid phobia.^5^

IPL technology has appeared for many years with different indications for multiple skin diseases. There are reports on the treatment of vascular diseases and pigmented lesions. Nevertheless, since laser treatments have become very popular among patients and physicians, IPL treatments have become less eye‐catching. Our team has been focusing on IPL technology for 20 years and we hope our reports of treating severe conditions ^6^, ^7^ can drive physicians to do more research on this valuable technology. The 675 nm laser is emerging as a safe and effective option for treating acne scars, particularly in individuals with darker skin types. Unlike ablative lasers, which are more aggressive and can cause hyperpigmentation, the 675 nm wavelength targets collagen fibers without affecting the vascular components of the dermis. This specificity reduces the risk of adverse effects, such as skin peeling, erythema, and hyperpigmentation, making it suitable for darker phototypes. Furthermore, the integrated skin cooling mechanism protects the epidermis, enhancing the safety profile of the treatment.^8^, ^9^, ^10^ IPL was shown to be effective in the treatment of hypertrophic scar. Potential complications with IPL include pain, burn, and post‐inflammatory hyperpigmentation.

## CONFLICT OF INTEREST STATEMENT

We acknowledge that we have considered the conflict of interest statement included in the “Author Guidelines.” We hereby certify that, to the best of our knowledge, no aspect of our current personal or professional situation might reasonably be expected to significantly affect our views on the subject we are presenting.

## Data Availability

The data that support the findings of this study are available from the corresponding author upon reasonable request.
